# Traffic-Related Air Pollution and Child BMI—A Study of Prenatal Exposure to Nitrogen Oxides and Body Mass Index in Children at the Age of Four Years in Malmö, Sweden

**DOI:** 10.3390/ijerph15102294

**Published:** 2018-10-19

**Authors:** Kasper Frondelius, Anna Oudin, Ebba Malmqvist

**Affiliations:** Occupational and Environmental Medicine, Department for Laboratory Medicine, Lund University, 221 85 Lund, Sweden; kasper.frondelius@med.lu.se (K.F.); anna.oudin@med.lu.se (A.O.)

**Keywords:** air pollution, nitrogen oxides, obesity, overweight, children

## Abstract

Traffic-related air pollution could be a danger to the health of children. Earlier studies have linked prenatal exposure to an increased risk of a range of diseases and negative health outcomes, including overweight and obesity. Presently, a knowledge gap exists in investigating the risk of overweight and obesity among children exposed to lower levels of air pollution in utero. This study aimed to investigate the relationship between prenatal traffic-related air pollution (nitrogen dioxides (NO_x_) and traffic density) and childhood overweight and obesity in Malmö, Sweden. A cohort, based on attendance of a four-year check-up examination at Swedish Child Health Care (CHC) centers, and a parent-assessed questionnaire provided data on body-mass index adjusted for four-year-old children (ISO-BMI) as well as socioeconomic and health variables. We estimated exposure by using traffic density and levels of NO_x_ at the maternal geocoded residential level. Analysis of 5815 children was performed using binary logistic regression models. This study showed no associations of increased risk for childhood overweight or obesity through to prenatal exposure to NO_x_ in this low-exposure setting. We further suggest analysis of risks related to exposure levels ranging between the ones presented here and those proposed in previous literature.

## 1. Introduction

The increase of childhood overweight and obesity in Europe has plateaued during the last few years [1]. However, the present rates are still high and impose an increased risk for the development of several chronic diseases, such as cardiovascular disease and type 2 diabetes [2], as well as psychosocial problems, including lower self-esteem and depression [3]. The preschool age has been proposed as a critical period of development in a child’s life, and overweight and obesity during this time often persist into adulthood [4,5]. Indeed, childhood and adolescent overweight and obesity are key predictors of the probability of developing overweight and obesity as an adult [6]. Comorbidities related to childhood overweight are often similar to adult overweight and obesity, including high blood pressure and type 2 diabetes. Early life factors such as limited breastfeeding, exposure to smoking, and low birth weight alongside parental attitudes on health lifestyles and socio-economic status, increase the risk of child obesity [7,8]. Causal mechanisms behind associations are likely related to both social and biological factors [7,9].

Traffic-related air pollution, most often assessed as particulate matter (PM), have previously been linked to the development of overweight and obesity in different settings and at varying exposure levels. Possible mechanisms have been suggested through rodent-model studies, indicating exposure-related increase in inflammatory processes and visceral adiposity through cytokine and macrophage activation [10]. Air pollution exposure during early childhood and its impact on weight have previously been studied epidemiologically by Dong et al., Jerrett et al., Mao et al., and Fioravanti et al. [11,12,13,14]. Mao et al. and Fleisch et al. have investigated similar associations during the gestational period [12,15]. However, few studies have looked at the effect of gestational exposure to nitrogen oxides (NO_x_), a common traffic pollutant, and subsequent development of overweight and obesity among preschool children in low to medium exposure settings. Thus, this study aims to investigate the associations between prenatal exposure to traffic-related air pollution and overweight and obesity among four-year-old children in Malmö, Sweden. The hypothesis was that exposure to traffic-related air pollution is associated with higher risk of overweight and/or obesity.

## 2. Materials and Methods

### 2.1. Population and Area of the Study

**Figure 1:**
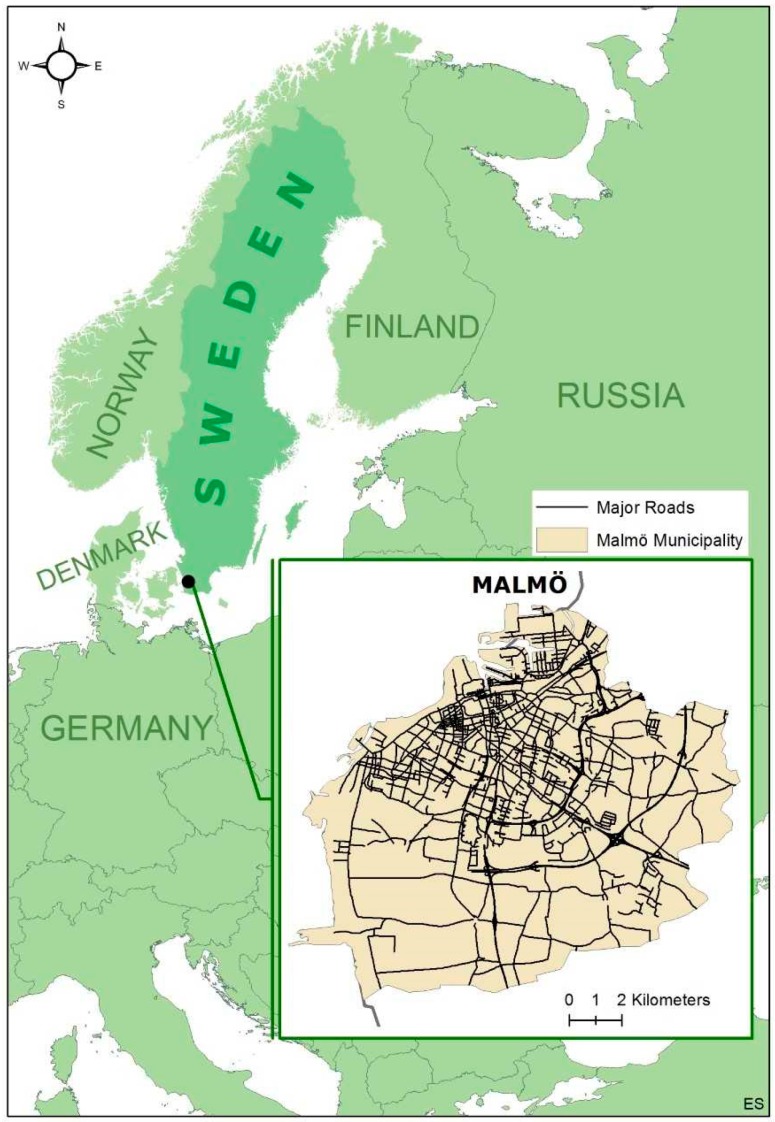
Map of the study area, Malmö, and its location in Sweden and Europe. In the inserted picture, the major road network of Malmö is highlighted. Map produced by Emilie Stroh.

The research setting is the city of Malmö (see Figure 1) located in the southern part of Sweden with a population of 300,000. Malmö tends to have higher levels of air pollution compared to other cities in Sweden [16,17]; however, the levels are generally below the thresholds set by the World Health Organization (WHO) and European Union [18]. The city is a melting pot representing various cultures, ethnicities, and languages with one third of its inhabitants born outside the borders of Sweden [19].

In Sweden, Child Health Care (CHC) centers invite all four-year-old children for a check-up. Being free of charge, CHC centers cover up to 99% of the Swedish child population, making their records a solid source from which to collect data [20]. The focus of this four-year examination is to assess child growth (weight and height), dietary habits, physical activity, and socioeconomic status (SES); to test both cognitive development and that of sight and hearing; and to screen for psychological disorders [7]. Researchers from Lund University provided questionnaires, which were then administered by pediatric nurses at the CHCs in Malmö. The questionnaire inquired about child and parental health, SES, and lifestyle-habits. For increased representation, the questionnaire translated into five languages: Albanian, Arabic, English, Serbo-Croatian, and Somali. From the derived information, researchers could examine underlying causes of ill health. Other studies in Sweden have used data collected from the questionnaires; further described the validation process of this questionnaire [7]. As per standard ethical guidelines, all parents gave their informed consent for participation in this study, which was approved by the Regional Ethical Committee in Lund (identification code 2014/696).

**Figure 2:**
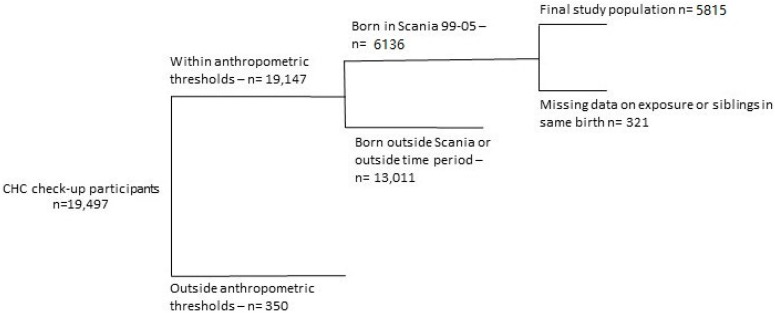
Flowchart of inclusion and exclusion criteria for final study population (n).

Children eligible to enter the study included those who underwent CHC centers’ four-year health check-up in Malmö and whose parents answered the aforementioned questionnaire (n = 19,497) (response rate 68%). Inclusion of children was further limited to anthropometrical data inside WHO’s scope of child growth standards for age 4–4.5, consisting of age-associated median height/weight ± three standard deviations (SD). Cut-off values for height were 90 and 120 cm, and 10 and 26 kg for weight [21], resulting in a population of n = 19,147. We also set inclusion criteria to children born in the county of Scania, where Malmö is located, from 1999–2005 to be able to assess perinatal exposure (n = 6136), as no exposure levels from outside the county were obtained. We further excluded children whose exposure data could not be acquired, such as those without a residential address registered in Statistics Sweden, as well as siblings from the same birth (i.e., twins, triplets). These criteria granted a final study population of 5815 children. For more details, see flowchart in Figure 2.

### 2.2. Assessment of Exposure

Using the 10-digit personal identification number (unique to every Swedish resident), we collected geocoded residency data from population-based registers at Statistics Sweden. The maternal residential address, is updated on a yearly basis, but no date on time of moving are available. Thus, if an individual did change addresses during pregnancy, her exposure was calculated based on the residential address nearest in time; for Jan–June we calculated exposure on prior address and for July–December we calculated exposure for the new address, (given that she still was residing in Scania). Sensitivity analyses excluding mothers who had moved address was performed.

We calculated each month of the pregnancy separately and aggregated to trimesters.

We then linked geocoded residential address of each mother to modelled levels of NO_x_. Exposure to NO_x_ was assessed using an emission database (EDB) for the region with emission information from approximately 24,000 sources. They were then divided into line sources, such as road traffic, shipping, and railway; point sources, including industries and large scale heating plants; and area sources, including aviation, construction machinery, and small-scale heating facilities. These sources were finally combined into a total level of NO_x_ exposure [16,22]. Due to atmospheric patterns and predominant wind patterns from the West pollution carried from Denmark over the Öresund Strait were included in the EDB [23]. The EDB was combined with a modified Gaussian dispersion model, AERMOD, taking both meteorology and height of emission source into consideration when accounting for incoming pollution [24]. However, local geographical features, such as street canyons, were not included in the model. An additional 2.5 µg/m^3^ was included, corresponding to measured annual background pollution levels at the regional background station, Vavihill. The fit of our model compared to previous testing was R^2^ = 0.69 [25]. Our model has also been tested comparing hourly mean NO_x_ values modelled into spatial 100 × 100 m areas and measured façade concentrations, which have correlated with Spearman’s correlation coefficient 0.8, n = 142 [26]. We have previously used the same exposure assessments regarding prenatal exposure in other epidemiological studies on prenatal exposure [27,28,29]. Concentrations (µg/m^3^) of NO_x_ were modelled as monthly mean values in spatial cells of 500 × 500 m, a spatial resolution earlier shown as accurate and applicable when assessing monthly means [25]. We have applied the model in previous epidemiologic studies with good results [27,28]. We assessed and aggregated pregnancy exposure data into gestational means: trimester 1, 2, and 3, corresponding to pregnancy months 1 to 3, 4 to 6, and 7 to delivery, respectively [25,26,28,29]. Trimester-specific quartiles were calculated, with cut-offs being 13.64, 20.87, 27.13 µg/m^3^ for trimester one; 12.92, 20.54, 27.12 µg/m^3^ for trimester two; and 12.54, 19.77, 26.34 µg/m^3^ for trimester three. We calculated mean gestational (pregnancy) exposure of NO_x_ (µg/m^3^) as a mean of all trimester values. Quartiles for full pregnancy mean exposure to NO_x_ were also established, with cut-offs at 13.66, 21.27, and 26.56 µg/m^3^.

### 2.3. Assessment of Outcome

Trained nurses at the CHC centers measured the height and weight of the children at the time of the four-year check-up. Obesity and overweight status of the children was established by using body-mass index adjusted for Swedish children (ISO-BMI), defined in kg/m^2^ [30]. Cut-off values for ISO-BMI were established according to guidelines by Cole et.al [31,32]. ISO-BMI values were set to 17.55 kg/m^2^ and 19.29 kg/m^2^ for boys and 17.28 kg/m^2^ and 19.15 kg/m^2^ for girls, corresponding to adult overweight and obesity-standards (BMI >25 and >30 kg/m^2^, respectively). Underweight among the children was defined as ISO-BMI, cut-offs were 14.43 kg/m^2^ for boys and 14.19 kg/m^2^ for girls, which corresponds to recommended adult BMI standards <18.5 kg/m^2^.

### 2.4. Covariates

Based on a priori knowledge [12,33] a selection of covariates was used in this study: child parity, maternal and paternal BMI, socioeconomic status (as defined by frequency of economic stress, maternal and paternal level of education, country of origin and crowded living), smoking during pregnancy, exposure to passive smoking, breastfeeding, and sex [7,12,33]. Parental BMI, assessed from the questionnaire and treated separate for mothers and fathers, was divided into underweight (BMI <18.5 kg/m^2^); normal weight (BMI >18.5, ≤25 kg/m^2^); overweight (BMI >25, <30 kg/m^2^); and obesity (BMI >30 kg/m^2^). We excluded children whose parents’ weight was lower than 45 kg or higher than 200 kg, as well as those whose parents’ height measured under 140 cm or over 210 cm. Parental education level, collected separately for mothers and fathers, was aggregated into three groups: Primary (≤9 years of education), High school graduates, and University attendees (both graduates and non-graduates). Parental heritage and country of birth was categorized into three groups: “Both parents born in Sweden”, “One parent born abroad”, and “Both parents born abroad”. Economic stress was included and assessed with the question, “How many times during the past year did you lack enough money to afford the food or clothes of need to you and your family?” with possible answers: “Every month”, “Six months/year”, “Occasionally”, and “Never”. Another measure used to capture SES was Crowded living, defined as number of individuals living per room, which was divided into two groups: ≤2 or >2 individuals per room. Rough measures of child dietary exposure were collected from parents assessing the intake frequency of sweetened beverages, with available options being “Every day”, “If child wakes up at night”, “Once or twice per week”, “Never”, and “Don’t know”, which was further dichotomized into Yes and No. Duration of breastfeeding was categorized into intervals of 6 months: 0–6 months, 6–12 months, 12–18 months, 18–24 months, 24–30 months, and >30 months. Additionally, we included a variable capturing tobacco smoke exposure in the child’s daily environment. Passive smoking was reported with available responses of No; Yes, mother/stepmother smokes daily (also includes smoking outdoors); Yes, father/stepfather smokes daily (also includes smoking outdoors); and Yes, sibling, daily caretaker, or other person smokes daily (also includes smoking outdoors). We further categorized the passive smoking variable into Yes, No, and Data missing. 

We also used the following variables from Perinatal Revision Syd (PRS), Scania’s regional birth registry. From PRS we used parity was defined as the child’s order of birth and divided into three groups (1, 2 or >3), with adjustments for extreme values. For instance, parity >18 was set as missing, while five cases with parity from 12–18 were retained. Maternal smoking during pregnancy was also included in PRS with the response selections Yes, 1–9 cigarettes/day; Yes, 10 or more cigarettes/day; No, and was then grouped into Yes, No, and Data missing. The sex of the child was set as per their sex assigned at birth in PRS. 

### 2.5. Statistical Analyses

We performed analyses using logistic regressions comparing strata of obesity status. Normal weight was set as the reference, while overweight and obesity were pooled into a single category overweight/obesity. Analyses for underweight were conducted separate from overweight/obesity analyses. We performed analyses for trimester-specific and full pregnancy exposure, using continuous exposure in addition to dividing exposure into quartiles. For analysis, the three models described below were used for both NO_x_ and traffic density:

1. Crude model
2. A Basic model adjusting only for parental BMI and sex of the child
3. A Full model with additional adjustments for intake of sweetened beverages, smoking during pregnancy, smoking in child’s environment (passive smoking), parity, maternal education, paternal education, crowded living, breastfeeding, parental country of birth, and economic stress. Multi-collinearity between smoking variables was low (Pearson’s correlation coefficient 0.32, *p* < 0.05), thus we included both variables

Furthermore, we performed sensitivity analysis stratified by maternal BMI-status. Statistical significance was set to 95% for all analyses. Data was analyzed using IBM (SPSS Statistics, version 22.0. Chicago, IL, USA).

## 3. Results

### 3.1. Background Data

Background characteristics, prevalence data, and descriptive statistics for exposure and effect according to child-obesity status are presented in Table 1, along with crude odds ratios (OR) for covariates. Both full pregnancy mean and trimester-specific mean NO_x_-exposure data can be considered to be normally distributed (−2 < skewness < 2). Mean ISO-BMI for boys and girls was 16.1 and 15.9 kg/m^2^, respectively. In total, 12% of the children were overweight and 3% were obese. The boys showed lower rates of representation in all non-normal weight groups, where 5% of the boys were overweight and 1% obese, while overweight girls represented 14% and obese 3% of all girls. Nine percent of both boys and girls were underweight.

### 3.2. NO_x_ Exposure and ISO-BMI

Crude, basic, and fully adjusted models for associations between NO_x_ and underweight and pooled overweight/obesity status among four-year-olds are presented in Table 2. NO_x_ exposures for neither full pregnancy nor specific trimesters seem to be associated with overweight/obesity (odds ratios close to one). For example, the odds ratio (OR) for overweight/obesity associated with the fourth quartile (compared to the first quartile) of full pregnancy mean of NO_x_ was 1.03 with 95% confidence interval (95% CI) of 0.75–1.41. There is a tendency for odds ratios from the underweight category to be associated with gestational NO_x_ exposure, both for full pregnancy and specific trimesters. This holds true for virtually all quartiles of exposure, especially for quartile 4 in the fully adjusted model. However, the results were not statistically significant (Table 2). Stratifying the data by maternal weight status (underweight, normal weight, and pooled overweight and obesity) did not show any significant results (data not shown).

## 4. Discussion

We found no marked associations between traffic-related air pollution (NO_x_) exposure during fetal life and overweight and obesity at age four in Malmö, Sweden, an area with low to medium pollution levels, averaging around 20 µg/m^3^ between 1999 and 2005. These detected levels are far below levels recommended by WHO (<40 µg/m^3^) [34]. Furthermore, we found weak evidence of an association between NO_x_ and underweight.

A study on mice supported an association between prenatal air pollution and obesity formation by “programming” offspring for increased susceptibility to diet-induced weight gain [35]. Previous epidemiological research on the topic is scarce, regarding both gestational exposure of NO_x_ and overweight and obesity at age four. For gestational exposure to pollution, Mao et al. have reported increased risk of childhood overweight and obesity due to PM_2.5_ exposure in utero and throughout the first two years of life. Moreover, Rundle et al. described increased risk of childhood overweight and obesity at age five and seven from gestational exposure to polycyclic aromatic hydrocarbons (PAHs) [36]. Fleisch et al.’s study also showed lower birthweight and increased weight gain during the first six months of life for infants prenatally exposed to high-density traffic and closer roadway proximity [15]. Our results are, however, in line with another European study by Fioravanti et al., which found no overweight associations between either gestational or early childhood exposures to air pollution [13]. It is also in accordance with another Italian study that found no association between traffic volumes outside schools and ponderal excess (a measure of corporal composition) [37]. For exposure during early childhood, Jerrett et al. reported increased risk of obesity formation among children, ages 5–11, using comparable exposure models to this study. Dong et al. also reported increased risk for overweight among children due to traffic-related air pollution (both nitrogen dioxide and PM), when exposed during early childhood [14]. Compared to previous studies, ours is set in a low-exposure setting, which might explain the lack of association.

Certain features of this study limit the transferability of the results and comparability to others, such as the varying concentrations of air pollution exposure across the city of Malmö, often reflecting socioeconomic differences. For instance, high traffic-related air pollution levels can be seen in the lower strata of socioeconomic status [22]. Furthermore, the low air pollution levels represented in our study area limit comparability of results, as many studies are conducted where higher exposure levels exist. It has also been more common to study overweight associations of particles (PM) or PAHs than NO_x_. We had unfortunately no data on particles or PAHs for this data set and this is a limitation. NO_x_ is instead often used in the literature as a marker of of freshly combustion- emitted ultrafine particles [38]. Previous studies have found that there is a high correlation between NO_x_ and nitrogen dioxide (NO_2_) in most areas in Europe [39]. NO_2_ is also considered a good marker for the complex mixture of traffic-related air pollution [39]. We can’t, however, rule out that we could have found an air pollution effect if we have had exposure data on particles.

Although the statistical significance of our results is generally poor, a few findings do appear. Namely, an increased risk for underweight can be seen within the crude and basic models, along with a few scattered discoveries of increased risk for overweight and obesity among different levels of exposure. Some of these may be due to chance, while others may be due to true associations. Failure to adjust for confounding is also possible. There are, for example, indications that traffic noise could be associated with obesity, at least in some population groups [40]. Also plausible is that the low exposure levels of NO_x_ present in this study do not have a major impact on obesity development in childhood.

One weakness of the study is that exposure was only assessed at residential address. This is to-date standard procedure in air pollution epidemiology even though it is well-known that personal exposure levels and home address-bound levels have low accuracy [26]. This is likely because it does not account for exposure to traffic during work, commuting or other outdoor activities. Air pollution at residency is, however, a commonly used tool in epidemiological research [41]. In fact, we have used that approach in many previous Malmö-specific studies, with associations detected [27,28,29]. Data on dietary exposure was also weak, consisting of only one question on intake of sweetened beverages. We also lacked data on other suggested variables, such as maternal BMI at conception and exposure to traffic noise [12,42]. Additionally, there is no information present regarding physical activity in the family, an effect which may be influenced and masked by having overweight or obese parents [7]. Air pollution in heavily polluted areas has been shown to discourage physical activity [43], but information on this trend in areas with lower pollution levels is less known. The lack of some possible confounders such as dietary habits, physical activity, access to green areas, noise and sleeping habits could have skewed our results towards null associations.

The main strength of the present study is the large dataset from the CHC centers with the availability of adjusting for potential confounding covariates. Considering the high participation rate in the center’s four-year check-up, and the comprehensive translation of the questionnaire, a sample representative of the city population was collected, thus minimizing the risk of selection bias and increasing generalizability [7,20]. The individually modelled NO_x_ exposure also served as a study strength.

### Suggestions for Future Studies

We recommend additional studies to examine the relationship between NO_x_ and the development of child overweight and obesity, especially in areas where air pollution levels are low to moderate. We further suggest identifying plausible biological mechanisms of this development.

## 5. Conclusions

We found no consistent associations between fetal exposures to elevated levels of NO_x_ and increased risk for childhood obesity in this low-exposure area.

## Figures and Tables

**Table 1:** Background table of covariates in relation to ISO-BMI categories of underweight, normal, overweight and obese.

Variable (n (%))	Underweight	OR	Normal Weight	Overweight	OR	Obese	OR	Total
**Total**	532	-	4430	705	-	148	-	5815
**Maternal BMI**								
<18.5	16 (13)	1.50	92 (77)	7 (6)	0.60	4 (3)	1.90	119
≥18.5, <25 ^1^	94 (9)	-	2528 (78)	344 (11)	-	59 (2)	-	3225
≥25, <30	107 (9)	1.08	851 (73)	172 (15)	1.50 *	32 (3)	1.60 *	1162
≥30	28 (6)	0.75	320 (73)	72 (16)	1.70 *	21 (5)	2.80 *	441
**Maternal Education**								
<6 years ^1^	15 (10)	-	116 (76)	20 (13)	-	2 (1)	-	153
6–8 years	16 (11)	1.19	104 (72)	15 (10)	0.84	10 (7)	5.60 *	145
≤9 years	35 (7)	0.73	373 (79)	50 (11)	0.78	16 (3)	2.49	474
High school	201 (10)	1.06	1472 (74)	260 (13)	1.02	56 (3)	2.00	1989
University attendee –	73 (9)	0.92	612 (77)	99 (12)	0.94	13 (2)	1.23	797
University degree	150 (8)	0.78	1483 (78)	222 (12)	0.87	37 (2)	1.45	1892
**Pregnancy Smoking**								
No ^1^	489 (10)	-	3835 (77)	586 (12)	-	114 (2)	-	5024
Yes	31 (5)	0.50 *	485 (75)	103 (16)	1.40 *	28 (4)	1.90 *	647
Don’t know	1 (17)	1.57	5 (83)	0	0.01	0	0.01	6
**Breastfeeding**								
<6 months ^1^	109 (9)	-	932 (75)	163 (13)	-	33 (3)	-	1237
6–12 months	168 (9)	1.02	1410 (77)	207 (11)	0.84	44 (2)	1.50	1829
12–18 months	85 (9)	1.03	708 (77)	106 (12)	0.86	26 (3)	0.72	925
18–24 months	35 (10)	1.13	265 (75)	47 (13)	1.01	6 (2)	0.64	353
24–30 months	12 (8)	0.88	117 (75)	24 (15)	1.17	3 (2)	1.04	156
>30 months	9 (7.)	0.82	94 (76)	16 (13)	0.97	5 (4)	0.88	124
**Parental Origin**								
Both Sweden ^1^	242 (8)	-	2370 (77)	408 (13)	-	79 (3)	-	3099
One born abroad	94 (10)	1.23	752 (78)	105 (11)	0.81	19 (2)	0.76	970
Both born abroad	186 (11)	1.50 *	1215 (75)	78 (11)	0.85	47 (3)	1.16	1626
**Paternal BMI**								
<18.5	2 (10)	0.84	19 (91)	0	-	0	-	21
≥18.5, <25 ^1^	194 (10)	-	1554 (80)	175 (9)	-	31 (2)	-	1954
≥25, <30	179 (9)	0.93	1542 (74)	301 (15)	1.70 *	58 (3)	1.90 *	2080
≥30	43 (8)	0.90	382 (74)	69 (13.4)	1.60 *	22 (4)	2.90 *	516
**Paternal Education**								
<6 years ^1^	14 (12)	-	86 (75)	13 (11)	-	2 (2)	-	115
6–8 years	6 (5)	0.70	97 (75)	19 (15)	1.30	7 (5)	3.10	129
≤9 years	51 (9)	0.72	408 (75)	66 (12)	1.07	22 (4)	2.32	547
High school	203 (9)	0.76	1642 (75)	294 (13)	1.18	57 (3)	1.49	2196
University attendee	70 (9)	0.77	598 (79)	71 (9)	0.79	15 (2)	1.08	754
University degree	133 (9)	0.38	1172 (78)	178 (12)	1.01	25 (2)	0.92	1508
**Economic Stress**								
Every month	19 (9)	1.01	146 (68)	43 (20)	1.95 *	8 (4)	1.90	216
Half the months of the year	14 (9)	0.98	120 (75)	20 (12)	1.10	7 (4.	2.00	161
Occasionally	53 (8)	0.95	470 (74)	88 (14)	1.24	23 (4)	1.70 *	634
None ^1^	417 (9)	-	3503 (77)	528 (12)	-	101 (2)	-	4549
**Passive Smoking**								
Yes	111 (8)	0.86	1023 (75)	192 (14)	1.25 *	98 (2)	1.48 *	1371
No ^1^	404 (9)	-	3294 (77)	496 (12)	-	92 (2)	-	4292
**Parity of Child**								
1–2 ^1^	372 (9)	-	3236 (76)	525 (12)	-	105 (3)	-	4238
3–4	103 (10)	0.86	751 (75)	120 (12)	0.99	31 (3)	1.28	1005
≥5	17 (8)	1.20	173 (81)	19 (9)	0.68	4 (2)	0.71	213
**Crowded Living**								
≤2 ^1^	330 (8)	-	3013 (77)	479 (12)	-	94 (2)	-	3916
>2	174 (11)	1.40 *	1153 (74)	189 (12)	1.03	47 (3)	1.30	1563
**Intake of Beverages**								
Every day	60 (9)	1.14	479 (74)	87 (14)	1.05	19 (3)	1.41	645
At night, if awake	4 (11)	1.50	24 (67)	6 (17)	1.44	2 (6)	2.96	36
1–2 times/ week	379 (9)	1.08	3181 (77)	497 (12)	0.90	101 (2)	1.13	4158
Never ^1^	47 (8)	-	426 (76)	74 (13)	-	12 (2)	-	559
Don’t know	13 (9)	1.03	114 (78)	15 (10)	0.76	5 (3)	1.56	147

^1^ Reference group. * *p* < 0.05.

**Table 2:** Odds Ratios (ORs) and their 95% Confidence Intervals (95% CIs) of underweight and pooled overweight and obesity in association with trimester-specific quartiles of NO_x_ (Q1–Q4) —exposure in crude, basic and full models.

	Crude		Basic ^1^		Full ^2^	
	Underweight	Overweight and Obesity	Underweight	Overweight and Obesity	Underweight	Overweight and Obesity
	OR (95% CI)	OR (95% CI)	OR (95% CI)	OR (95% CI)	OR (95% CI)	OR (95% CI)
**Full Pregnancy** ^3^						
Q1	-	-	-	-	-	-
Q2	1.57 * (1.21–2.03)	0.86 (0.70–1.06)	1.49 * (1.11–1.98)	0.80 (0.63–1.01)	1.31 (0.91–1.89)	1.03 (0.76–1.39)
Q3	1.27 (0.97–1.65)	0.82 (0.67–1.01)	1.18 (0.87–1.59)	0.76 * (0.60–0.97)	1.03 (0.69–1.54)	0.87 (0.63–1.20)
Q4	1.15 (0.88–1.51)	0.95 (0.77–1.16)	1.15 (0.85–1.57)	0.99 (0.79–1.25)	1.26 (0.83–1.93)	1.03 (0.75–1.41)
**Trimester 1** ^4^						
Q1	-	-	-	-	-	-
Q2	1.33 * (1.03–1.73)	0.98 (0.80–1.20)	1.27 (0.95–1.71)	1.00 (0.79–1.26)	1.03 (0.71–1.50)	1.28 (0.95–1.72)
Q3	1.29 (0.99–1.68)	0.88 (0.71–1.08)	1.24 (0.93–1.67)	0.78 (0.61–1.00)	1.06 (0.71–1.57)	0.92 (0.67–1.27)
Q4	1.19 (0.91–1.55)	0.85 (0.69–1.04)	1.12 (0.83–1.52)	0.88 (0.69–1.12)	1.21 (0.81–1.81)	0.93 (0.68–1.28)
**Trimester 2** ^5^						
Q1	-	-	-	-	-	-
Q2	1.29 (0.99–1.68)	0.94 (0.81–1.22)	1.26 (0.94–1.71)	1.02 (0.81–1.29)	1.01 (0.69–1.48)	1.31 (0.97–1.76)
Q3	1.30 * (1.00–1.69)	0.87 (0.70–1.06)	1.25 (0.93–1.67)	0.78 * (0.61–1.00)	1.09 (0.73–1.61)	0.90 (0.65–1.23)
Q4	1.18 (0.90–1.54)	0.85 (0.69–1.04)	1.13 (0.83–1.53)	0.88 (0.69–1.12)	1.21 (0.81–1.83)	0.92 (0.67–1.27)
**Trimester 3** ^6^						
Q1	-	-	-	-	-	-
Q2	1.33 * (1.01–1.75)	0.99 (0.80–1.22)	1.34 (0.99–1.82)	1.04 (0.81–1.32)	1.10 (0.75–1.63)	1.27 (0.94–1.73)
Q3	1.28 (0.98–1.67)	0.91 (0.74–1.12)	1.18 (0.87–1.59)	0.87 (0.68–1.11)	0.99 (0.66–1.48)	1.01 (0.74–1.38)
Q4	1.21 (0.92–1.57)	0.84 (0.68–1.03)	1.16 (0.86–1.57)	0.86 (0.68–1.10)	1.29 (0.87–1.93)	0.91 (0.67–1.25)

^1^ Adjusted for parental BMI and sex. ^2^ Adjusted for parental BMI, sex, smoking during pregnancy, passive smoking, breastfeeding, parity, intake of sweetened beverages, economic stress, crowded living, maternal and paternal education, birth year child, and parental place of birth. ^3^ Quartiles (Q) for full pregnancy mean exposure to NO_x_ cut-offs at 13.66, 21.27 and 26.56 µg/m^3^. ^4^ Quartiles (Q) for trimester one mean exposure to NO_x_ cut-offs at 13.64, 20.87, 27.13 µg/m^3^. ^5^ Quartiles (Q) for trimester two mean exposure to NO_x_ cut-offs at 12.92, 20.54, 27.12 µg/m^3^. ^6^ Quartiles (Q) for trimester three mean exposure to NO_x_ cut-offs at 12.54, 19.77, 26.34 µg/m^3^. * *p* < 0.05.
